# A Novel Hybrid Technique Combining Improved Cepstrum Pre-Whitening and High-Pass Filtering for Effective Bearing Fault Diagnosis Using Vibration Data

**DOI:** 10.3390/s23229048

**Published:** 2023-11-08

**Authors:** Amirmasoud Kiakojouri, Zudi Lu, Patrick Mirring, Honor Powrie, Ling Wang

**Affiliations:** 1 National Centre for Advanced Tribology at Southampton (nCATS), School of Engineering, University of Southampton, Southampton SO17 1BJ, UK; 2 Southampton Statistical Sciences Research Institute (S3RI), School of Mathematical Sciences, University of Southampton, Southampton SO17 1BJ, UK; z.lu@soton.ac.uk; 3 Schaeffler Technologies AG & Co. KG, Georg-Schaefer-Str. 30, 97421 Schweinfurt, Germany; mirriptr@schaeffler.com; 4 GE Aerospace, School Lane, Chandlers Ford, Eastleigh SO53 4YG, UK; h.powrie@soton.ac.uk

**Keywords:** REBs, vibration analysis, CPW, ICPW, envelope analysis, incipient fault diagnosis, multiple faults detection

## Abstract

Rolling element bearings (REBs) are an essential part of rotating machinery. A localised defect in a REB typically results in periodic impulses in vibration signals at bearing characteristic frequencies (BCFs), and these are widely used for bearing fault detection and diagnosis. One of the most powerful methods for BCF detection in noisy signals is envelope analysis. However, the selection of an effective band-pass filtering region presents significant challenges in moving towards automated bearing fault diagnosis due to the variable nature of the resonant frequencies present in bearing systems and rotating machinery. Cepstrum Pre-Whitening (CPW) is a technique that can effectively eliminate discrete frequency components in the signal whilst detecting the impulsive features related to the bearing defect(s). Nevertheless, CPW is ineffective for detecting incipient bearing defects with weak signatures. In this study, a novel hybrid method based on an improved CPW (ICPW) and high-pass filtering (ICPW-HPF) is developed that shows improved detection of BCFs under a wide range of conditions when compared with existing BCF detection methods, such as Fast Kurtogram (FK). Combined with machine learning techniques, this novel hybrid method provides the capability for automated bearing defect detection and diagnosis without the need for manual selection of the resonant frequencies. The results from this novel hybrid method are compared with a number of established BCF detection methods, including Fast Kurtogram (FK), on vibration signals collected from the project I2BS (An EU Clean Sky 2 project ‘Integrated Intelligent Bearing Systems’ collaboration between Schaeffler Technologies and the University of Southampton. Safran Aero Engines was the topic manager for this project) and those from three databases available in the public domain—Case Western Reserve University (CWRU), Intelligent Maintenance Systems (IMS) datasets, and Safran jet engine data—all of which have been widely used in studies of this kind. By calculating the Signal-to-Noise Ratio (SNR) of each case, the new method is shown to be effective for a much lower SNR (with an average of 30.21) compared with that achieved using the FK method (average of 14.4) and thus is much more effective in detecting incipient bearing faults. The results also show that it is effective in detecting a combination of several bearing faults that occur simultaneously under a wide range of bearing configurations and test conditions and without the requirement of further human intervention such as extra screening or manual selection of filters.

## 1. Introduction

Rolling element bearings (REBs) have been shown to have a high failure rate in rotating machinery. In 1986, an industry assessment study on power generation motors revealed that REB failures accounted for over 40% of the total breakdowns [1]. Moreover, other research showed that bearing failures account for 7% of all gas turbine engine failures and thus negatively affect fleet management [2]. Therefore, bearing Condition Monitoring (CM) can play an important role in reducing mechanical breakdowns and increasing the reliability of assets. For rotating machinery, Vibration-based Condition Monitoring (VCM) is one of the most effective CM techniques due to its demonstrated ability to detect abnormalities before catastrophic failures occur in machines [3].

As a result of a localised fault in one of the bearing components (i.e., inner race, outer race, and ball or roller), impulsive signatures are generated due to the rolling contact. This leads to periodic impulses appearing in the time domain vibration signals and the corresponding bearing characteristic frequencies (BCFs (BCFs include ball pass frequency outer race (BPFO), ball pass frequency inner race (BPFI), ball spin frequency (BSF), and fundamental train frequency (FTF))) may be seen in the frequency spectra. However, at the incipient stage, the amplitudes of the BCFs may be relatively low compared with other sources of noise and vibration within the machine, such as those from a drivetrain or gear systems, and thus it is challenging to readily detect BCFs. Envelope analysis [4], also known as the High-Frequency Resonance Technique (HFRT), is a powerful tool, especially in industrial practices. It is primarily utilised for amplitude demodulation, aiming to distinguish the high-frequency resonant signal and the BCF signal component from noise and other interferences. This technique is essential for overcoming interferences from other sources and noise in the machine. One of the key steps when using HFRT is that each raw vibration signal is firstly filtered by a set of band-pass filters within a pre-determined resonance region, excited by the particular bearing fault, in order to distinguish BCFs [5]. Selection of a suitable band-pass filter has been challenging in real applications due to the instantaneous nature of the bearing resonant frequencies, especially when BCFs are weak, i.e., when faults are at the incipient stage [6]. This is because impulses caused by an incipient bearing fault can excite a range of natural frequencies in an REB system associated with bearing races, rolling elements (ball or roller), housings, or any other part of a machine. Thus, choosing the correct band-pass filter is one of the major challenges in the application of HFRT, predominantly due to the intensive manual screening of possible filters [7]. To add to the complexity, the natural frequencies in one machine excited by impulses of a bearing fault may also vary due to the changing operating conditions and/or other faults in the system. To date, in many real-world applications, filter selection has been predominantly conducted by experts who look through the spectra manually [8]. Moreover, discrete vibration frequencies in signals caused by shaft-related faults and gear meshing, are typically at higher amplitudes compared with those generated by REB faults and thus often mask BCFs [9]. Over the past two decades, a number of signal processing techniques have been developed to select the most suitable filters for effective bearing fault diagnosis, which are discussed in three categories below.

The first category of these techniques uses ‘decomposition’ of the original vibration signal, i.e., produces multiple sub-signals that each include a specific frequency mode. Common examples from this category are Empirical Mode Decomposition (EMD), Ensemble Empirical Mode Decomposition (EEMD), Empirical Wavelet Transform (EWT), and Variational Mode Decomposition (VMD). The main limitation of these approaches is that EMD is susceptible to mode mixing, while EWT and EEMD necessitate parameter tuning and the selection of appropriate levels. Also, it is often required to pre-determine the number of sub-signals to enable the selection of the most useful sub-signal for the envelope and hence, bearing fault detection [10,11]. These factors limit their effective use in practical applications. 

The second category of techniques for band-pass filter selection involves the application of a series of filters to the signal to determine the optimum band for the HFRT. Examples of methods from this category include Continuous Wavelet Transform (CWT), Spectral Kurtosis (SK), and Kurtogram. These techniques also require parameter optimization (different from those in the decomposition category) to determine the frequency band. For example, in CWT, after an initial wavelet is selected, parameters such as scale and bandwidth have to be selected to determine the optimal frequency band for HFRT [12]. This is further complicated by the fact that there are initially numerous wavelets to be selected, which significantly increases the cost of computation and thereby reduces the attractiveness of this method [13,14]. In the SK and Kurtogram techniques, an optimisation process is conducted to maximise the kurtosis of the filtered signal [15]. Due to the high sensitivity of the kurtosis index to random noise and aperiodic impulses occurring in most real applications, it is challenging to apply these methods for the detection of bearing faults in industrial scenarios [13]. In a separate investigation, scientists aimed to address the problem associated with the SK using kurtosis-guided-grams based on the Gini index [16] for diagnosing bearing faults. Nevertheless, this approach necessitates a baseline signal from the machine in a healthy condition, which is a requirement that may not always be feasible in industrial settings.

The third category of techniques is based on eliminating discrete or deterministic components in vibration signals. These signals are due to other parts of the machine such as eccentricity, bending, and misalignment of the rotating shaft, as well as gear meshing, and typically have relatively high vibration amplitudes, thus masking BCFs and making bearing fault detection more challenging. These discrete components are different from the signals caused by bearing faults and can be described by single parameters of amplitude, frequency, or oscillation rate [9]. Examples of techniques in this category include Time Synchronous Averaging (TSA), Adaptive Noise Cancelation (ANC), and Cepstrum Pre-Whitening (CPW). TSA is one of the best methods for the removal of discrete frequency components; however, it requires prior knowledge of each set of harmonics present in the signal [17]. ANC was originally proposed as a technique to extract BCFs in a signal from a faulty bearing in a gearbox, where the primary signal was obtained from a sensor on the faulty bearing, and a reference signal was obtained from a sensor located remotely from the faulty bearing to eliminate noise influences. However, this approach would not be possible for a planetary gearbox since signals must be transmitted through the ring gear in a planetary gearbox [18]. To overcome the limitations of ANC, a self-adaptive noise cancellation algorithm (SANC) was introduced using extra sensors that track the order of the vibration signals to avoid long time consumption for its convergence [18,19]. While CPW has been recognised as a powerful technique for removing discrete components and machine resonant frequencies from vibration signals [20], its ability to detect incipient bearing faults is found to be lessened by significant background noise [21,22]. 

In summary, many methods have been developed over the years, including those developed in recent years [23,24,25,26,27,28,29,30,31]; however, there is no simple solution for the selection of an effective band filter for the HFRT for real-world applications. Moreover, the application of machine learning techniques in this domain has attracted a significant amount of attention due to their potential to enhance diagnostic accuracy and efficiency. Recent research in this area, as highlighted in [31,32,33], has shown promising results in machine learning models for bearing fault diagnosis, including using Continuous Wavelet Transform (CWT), spectrograms, and segmented time-frequency representations as pre-processing techniques to prepare input data for convolutional neural networks (CNNs). These approaches have yielded significant enhancements in fault detection accuracies. Nevertheless, they also present specific limitations and issues, such as challenges associated with CWT that were identified. More notably, they highlighted a crucial limitation, which is the lack of generalizability. This issue manifests when the training and testing data distributions coincide, i.e., these models are unsuitable for testing on new machine data due to their inability to adapt to varying data sources.

This paper presents a novel hybrid method combining CPW and high-pass filtering methods with the aim to detect BCFs by overcoming the shortcomings of existing methods, including the removal of manual steps, and thus moving towards automatic bearing fault detection. The rest of this paper is organised as follows. Section 2 introduces the theoretical background of CPW and describes the novel hybrid method in detail. Section 3 describes the creation of numerically simulated vibration signals, based on the IMS data with defects [34], which are then used to verify the novel hybrid method. Section 4 validates the novel hybrid method using experimental data from four sources, including our own studies in I2BS [35,36] and publicly available datasets such as CWRU [37], IMS, and the Safran jet engine challenge [38]. A comparison study between the novel hybrid method and a well-established method FK is conducted and the results are summarised in Section 5. Finally, conclusions from this study are presented in Section 6.

## 2. Methodology

### 2.1. The Basic Theory of Cepstrum Pre-Whitening (CPW)

CPW is based on cepstrum analysis and is one of the most powerful techniques for removing discrete components from a vibration signal. Cepstrum analysis has several versions and was originally defined as the “power spectrum of a logarithmic spectrum”, whereas the latest version of it is defined as the “inverse Fourier transform of a logarithmic spectrum”, which is described by Equation (1) [39].

(1) $$C=IFT\left(log\left|FFT\left(x\right)\right|\right)$$

where x represents the vibrational waveform and C is the cepstrum of x. This technique is useful in identifying periodic (discrete) components. Second-order cyclostationary signals, such as those due to bearing faults, do not exhibit strong peaks in the absolute value of the corresponding cepstrum. Based on the cepstrum, CPW has been developed to further weaken discrete components, thus highlighting impulsive signatures in the signals. In CPW, the whole real part of the cepstrum is zero value (except that at zero quefrency), which is then recombined with the original signal phase and transformed into a time-domain signal. This procedure is equivalent to a series of liftering that is equivalent to filtering in spectra, which operates around frequencies of periodic excitations and results in the almost complete removal of their influence and the elimination of resonance frequencies in signals. Instead of cepstrum domain analysis, a simpler method of implementing CPW is to divide the Fourier transformed signal by the absolute value and transform the result back to the time domain, as shown in Equation (2) [39].

(2) $$x_{cpw}=IFT\left\{\frac{FFT\left(x\right)}{\left|FFT\left(x\right)\right|}\right\}$$

In this approach, since all spectral bands are normalised to have the same power density, the ones with greater impulsivity, e.g., due to bearing faults, tend to dominate the waveforms [39]. Although this technique has relatively low accuracy in noisy conditions and with incipient faults, it is straightforward to implement and does not require complicated analysis or manual derivative of parameters.

### 2.2. The Novel Hybrid Method

Due to the difficulties that CPW faces in detecting fault characteristics of rolling element bearings at the incipient stage and under noisy conditions, a novel hybrid method combining an improved CPW and the enveloping of high-pass-filtered signals is developed in this study, taking advantage of both methods. 

The improved CPW (ICPW) combines basic CPW and filtering of the signal from CPW using a bank of band-pass filters in an automated manner. The difference is that in basic HFRT, a resonance frequency band has to be selected empirically to identify BCFs with the effective removal of frequency components that are not related to bearing fault features, while the ICPW uses a bank of band-pass filters over the whole frequency range instead of the single band-pass filter in HFRT. The sum of the spectra obtained from the band-pass-filtered signals in the ICPW provides a single spectrum at the end of the procedure. By taking the whole frequency range into account, all frequency bands have a chance to contribute to the fault diagnosis. Bearing defect frequencies can thus be highlighted, as the ICPW could potentially remove frequencies related to shaft rotating frequency and harmonics and other discrete components.

Similar to CPW, the ICPW may weaken fault features due to background noise. In cases where defect frequencies are extremely weak, a high-pass filtering method can remove low frequencies related to shaft rotating harmonics and other noise, thus improving the detection of bearing defects [40]. It is thus proposed that a high-pass envelope be used together with the ICPW to eliminate the requirement of any optimisation or parameter selection procedure. 

**Figure 1:**
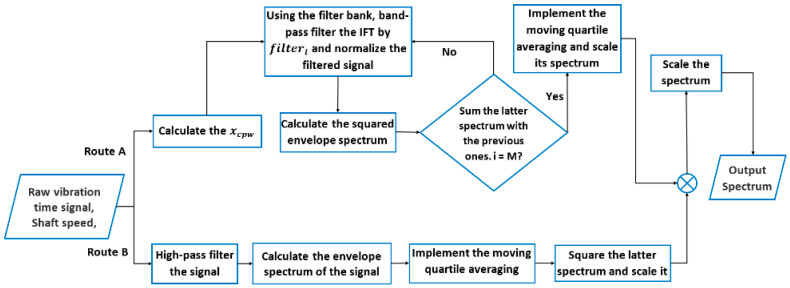
A flowchart of the novel hybrid method.

The novel hybrid method, illustrated in Figure 1, starts from a raw vibration signal, and goes through two parallel processes: the ICPW (route A) and high-pass filtering (route B), before the spectra are combined to produce one spectrum for BCF identification. Details of the processes are given below.

**Route A: ICPW**

- Calculate the $x_{CPW}$ of the original time domain vibration signal according to Equation (2) to remove the discrete components in the signal.
- Filter the $x_{CPW}$ signal with a bank of band-pass filters. The centre frequencies and bandwidth of the band-pass filters are defined based on the bearing dimensions and rotating speed. The bandwidth of the filters has been suggested to satisfy $\sigma >3\times f_{BCF}$ [41], where σ is the bandwidth of the original signal. In this study, a constant bandwidth value of $\sigma =5\times f_{BPFI}$ is chosen to simplify the process because the $f_{BCF}$ corresponds to BPFI, or $f_{BPFI}$, which is the largest among BCFs. The distance between two consecutive centre frequencies is kept at the shaft speed. In this step, M filters (${filter}_{I};i=1,\ldots M$) are designed so that the first filter’s lower cut-off frequency is equal to $35\times f_{shaft}$ to reduce the interference effects from shaft harmonics [42], and the last filter’s higher cut-off frequency is equal to $0.5\times f_{sampling}$. Thus, the whole available frequency range is swept. Each band-pass-filtered signal is then normalised to zero mean and unit variance using Equation (3).
  (3) $$x_{filtered\_normalized\_i}=\frac{x_{filtered\_i}-mean(x_{filtered\_i})}{std(x_{filtered\_i})}$$
  where std stands for the standard deviation operator.
- The normalised signal is then processed with Hilbert Transform (HT) and Fast Fourier Transform (FFT) to calculate the envelope spectrum. The envelope signal is then squared to highlight transient $f_{BCFs}$ components and their harmonics and to attenuate the stationary Gaussian noise in the spectrum [43].
- After all the band-pass filters are swept through, the sum of all the squared envelope signals is calculated.
- To reduce the background noise in the spectrum, a moving quartile function with a population of 0.85 and a window size of $f_{BSF}$ is applied. Then, the amplitudes of the squared spectrum are divided by the largest peak in the spectrum to scale all components between 0 and 1.

**Route B: High-pass-filtered envelope**

- The original vibration time domain signal is firstly filtered with a high-pass filter with a cut-off frequency at $35\times f_{shaft}$.
- The filtered signal is then processed with Hilbert Transform (HT) to find its envelope [44]. Then, the FFT of the envelope signal is calculated to obtain its spectrum.
- Same as the last step in Route A, the moving average function with the same setting is applied.
- The squared envelope spectrum is calculated and scaled between 0 and 1, similar to the fifth step in Route A.

The two enveloped spectra obtained from Routes A and B are then summed and scaled between zero and one in a similar way as described in the fourth step of Route B. Since the method in Route A is able to detect bearing faults in the presence of discrete components due to other phenomena such as gear mesh, while that in Route B is effective in detecting incipient bearing faults under noisy conditions, the proposed hybrid method has the potential to improve bearing fault detection under a wide range of conditions. The effectiveness of the novel hybrid method is demonstrated using both simulated and experimental data from various sources in the next sections.

Furthermore, to assess the diagnostic effectiveness of the novel hybrid method, the BCFs detected are compared with existing spectral analysis methods, including the full-band envelope, high-pass-filtered signal envelope, basic CPW envelope, and improved CPW (ICPW) envelope. Comparison methods include viewing spectral plots (as widely used in the literature) and a quantification based on the Signal-to-Noise Ratio (SNR). SNRs for each type of fault are defined in Equations (4)–(6) for the outer ring, inner ring, and ball spin faults, respectively. The SNR considers root mean square (RMS) values of the three primary harmonics of the associated BCF, e.g., BPFO, BPFI, and BSF, and one pair of the sidebands for each harmonic of the BPFI and BSF. The noise level is calculated based on the RMS of the spectral signal excluding all the BCFs and their sidebands. 

For BSFs, where the presence of exclusively even harmonics or a combination thereof is feasible, a similar procedure is performed, but considering up to six BSF harmonics. Summations of the adjacent two harmonics, e.g., the sum of 1st and 2nd harmonics produces the first BSF amplitude; the sum of 3rd and 4th harmonics produces the second BSF; and so on. The corresponding sidebands are identified for each harmonic (1st to 6th) and summed in a similar way to ${BPFI}^{*}$ to produce three values. BCFs and sidebands identified in the spectrum are excluded in the Signal-to-Noise Ratio (SNR) calculation based on Equations (4)–(6). To locate BCFs and sidebands in a spectrum, a number of steps are followed. First, the theoretical values of BCF orders are calculated based on their corresponding formula. Then, the primary (first) harmonic of each BCF within a 4% range around the expected BCF order is investigated. This range accounts for slips in bearings and fluctuation in shaft speed. The highest amplitude within this range is selected as the actual BCF. Subsequently, the second and third harmonic amplitudes are determined by multiplying the first BCF actual order by 2 and 3, respectively, and finding their corresponding amplitudes within a 2% range. The approach for detecting sidebands is similar to identifying BCF harmonics.

(4) $${SNR}_{BPFO}=20{log}_{10}(\frac{rms([{BPFO}_{harmonic\;1},\;{BPFO}_{harmonic\;2},\;{BPFO}_{harmonic\;3}])}{rms(Spectrum\;with\;no\;BCFAmplitudes)})$$

(5) $${SNR}_{BPFI}=20{log}_{10}(\frac{rms\left(\left[{BPFI}_{1}^{*},\;{BPFI}_{2}^{*},\;{BPFI}_{3}^{*}\right]\right)}{rms\left(Spectrum\;with\;no\;BCFAmplitudes\right)})$$

(6) $${SNR}_{BSF}=20{log}_{10}(\frac{rms\left(\left[{BSF}_{1}^{*},\;{BSF}_{2}^{*},\;{BSF}_{3}^{*}\right]\right)}{rms\left(Spectrum\;with\;no\;BCFAmplitudes\right)})$$

where ${BPFI}_{i}^{*}={[BPFI}_{left\;sideband\;of\;harmonic\;i},{BPFI}_{harmonicsi},{BPFI}_{right\;sideband\;of\;harmonic\;i}],\;i=1,2,3.$

## 3. Verification of the Novel Hybrid Method

In this section, the accuracy of the novel hybrid method is evaluated using simulated vibration signals from a bearing with multiple bearing faults (indicated by BCFs) as well as a shaft fault (indicated by shaft rotating frequency and its harmonics) and a gear fault (indicated by a discrete gear mesh frequency) based on the models presented in [45,46]. The equation in Equation (7) models a vibration signal from an REB with a defect on its outer race and one on its inner race, where, as a result of the fault impulses in the bearing system, four natural frequencies are considered in this simulation. Amplitude modulators for the four natural frequencies are randomly selected without any duplications.

(7) $${x\left(t\right)}_{bearing\_faults}=\left(\sum_{i=1}^{4}A_{b_{i}}e^{-\alpha \omega_{n_{i}}t}\sin\left(\omega_{d_{i}}t\right)\right)*\sum_{j=1}^{J}\delta \left(t-{jT}_{BPFO}\right)\;\;+\left(\left(\sum_{i=1}^{4}A_{b_{i}}e^{-\alpha \omega_{n_{i}}t}\sin\left(\omega_{d_{i}}t\right)\right)*\sum_{k=1}^{K}\delta \left(t-{kT}_{BPFI}\right)\right)\cdot \left(1+\sin\left(2\pi f_{rotating\;shaft}t\right)\right)$$

where $A_{bi}$ is the amplitude modulator with a randomness of 25% for the ith natural frequency, α is the resonance damping coefficient, $\omega_{ni}$ is the ith natural frequency of the excited structure, $\omega_{di}$ is the damped natural frequency for the ith natural frequency, δ is the Dirac-delta function to convolve with resonance responses to create the impulse train in the signal, J and K are the number of impulses in vibration signal with the period of $T_{BPFO}$ and $T_{BPFI}$, respectively, and $f_{rotating\;shaft}$ is the shaft rotating frequency. Moreover, a 10% of randomness for the repetition rate of bursts is considered due to the usual slip of the rolling elements and the cage (i.e., $T_{i}=iT+\delta T_{i};i=BPFO,BPFI)$ [47].

Five shaft harmonics and ten gear mesh harmonics are simulated using Equation (8) and Equation (9), respectively [48].

(8) $$x_{shaft\;harmonics}(t)=\sum_{m=1}^{5}A_{s}\cdot sin(2\pi {if}_{rotating\;shaft}t+\varphi_{s})$$

(9) $$x_{gear\;mesh}(t)=\sum_{m=1}^{10}A_{g}\cdot sin(2\pi {iN_{g}f}_{rotating\;shaft}t+\varphi_{g})$$

where m is the number of harmonics; $A_{s}$ and $A_{g}$ are the amplitudes of the shaft harmonics and the gear mesh, respectively, with 25% of randomness; $N_{g}$ is the number of teeth in the gear located on the same shaft; and $\varphi_{s}$ and $\varphi_{g}$ are the phases of shaft harmonics and gear mesh with random values between 0 and 2π. 

Combining the signal with bearing inner race and outer race faults Equation (7) with shaft harmonics Equation (8) and the discrete vibration from the gear mesh Equation (9), as well as a white Gaussian noise signal, the simulated signal is expressed below in Equation (10):

(10) $$x_{simulated}\left(t\right)={x(t)}_{bearing\;faults}+x_{shaft\;harmonics}+x_{gear\;mesh}+n(t)$$

where $n(t)\sim N(0,0.1^{2})$ is white Gaussian noise [49]. 

Signals simulated based on the bearing dimensions and test scenarios presented in the Intelligent Maintenance System (IMS) [34] are used to verify the effectiveness of the novel hybrid method. The parameters required in Equations (2)–(9) are listed in Table 1. Experimental conditions and technical details are summarised in Table 2 and Table 3. 

**Figure 2:**
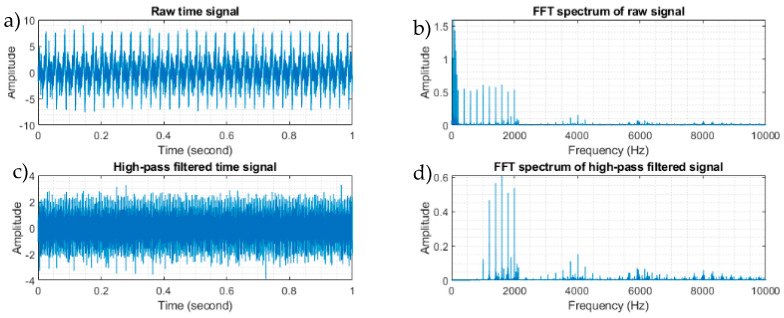
The simulated raw vibration signal with gear mesh components in the time (**a**) and frequency domains (**b**) and after being filtered using a high-pass filter of 1166.9 Hz in the time (**c**) and frequency domains (**d**).

**Figure 3:**
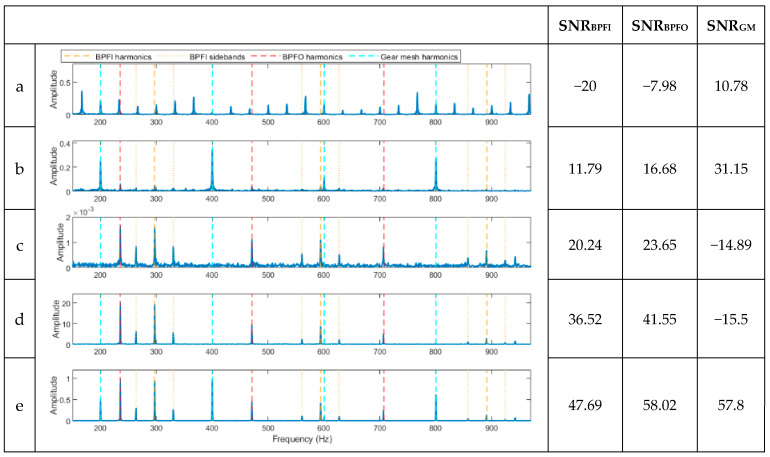
Results of the signal in Figure 2a after being processed using the proposed methods. (**a**) A spectrum of the full-band envelope. (**b**) A spectrum of the high-pass filtered envelope. (**c**) A spectrum of the basic CPW envelope. (**d**) A spectrum of the ICPW envelope. (**e**) A spectrum of the novel hybrid method.

Figure 2a,b illustrates the time and frequency plots of the simulated signal, while Figure 2c,d displays the frequency domain plots of the signal following filtration with a high-pass filter set at 35 times the rotating shaft frequency (equivalent to 1166.9 Hz). Clearly visible in Figure 2b are the discrete components linked to the shaft harmonics and gear mesh frequency. For fault diagnosis, five methods based on the envelope analysis were used on the processed signal: full-band envelope, high-pass filtered envelope, basic CPW, ICPW, and the novel hybrid method. The corresponding outcomes are depicted in Figure 3. Additionally, the SNR corresponding to each fault is presented in the right-hand columns of each spectrum.

As shown in Figure 3a, the full-band enveloping spectrum is completely dominated by the shaft harmonic frequency and its harmonics but does not detect any of the BCFs or gear mesh frequencies (GMFs). Since the gear mesh frequencies align with the shaft harmonics, the SNR value shown in Figure 3a does not accurately represent the SNR of the gear mesh frequencies. Next, after applying a high-pass filter to eliminate shaft harmonics, the envelope spectrum of the resulting signal is displayed in Figure 3b. The shaft harmonics are visibly absent, and the spectrum is dominated by the gear mesh frequencies. The BPFO and BPFI harmonics are faintly visible, exhibiting relatively low SNRs. On the other hand, the basic CPW does the opposite; it detects the harmonics of the BCFs and sidebands but not the gear mesh frequencies (Figure 3c) with noticeable background noise. ICPW, similar to CPW, detects the BPFO and BPFI with sidebands with low background noise (Figure 3d) but does not detect the gear mesh frequencies due to their discrete nature. This issue has been successfully resolved with the novel hybrid method, where the BPFO, BPFI, and gear mesh frequencies are all identified in one single spectrum (Figure 3e). 

To quantify the diagnostic results, the SNR values (shown on the right-hand side of the spectra in the figure) for the five analysis methods and the three faults are compared. For all three faults, i.e., inner ring, outer ring, and gear faults, the spectrum from the novel hybrid method shows the highest SNR values (47.69, 58.02, and 57.8, respectively) compared with the other four methods. 

The use of simulated signals with well-defined features demonstrates that the novel hybrid method can overcome the drawbacks of the other individual methods and has the capability of detecting weak BCFs in the presence of discrete signals, such as gear meshing in a noisy environment. The hybrid method can be implemented as a continuous (automated) process and, hence, without the difficulties of HFRT, where the most suitable resonance frequency band has to be manually defined for each signal sample. 

## 4. Validation of the Novel Hybrid Method with Experimental Data

To validate the effectiveness of the novel hybrid method, four case studies using experimental data are presented in this section. Each case presents different challenges for incipient bearing fault detection and includes noisy operating conditions. The first case study uses I2BS sub-scale test data, obtained in a parallel study by this research group, where bearings with seeded defects were tested under a wide range of conditions [50]. During these tests, a shaker was used to purposely add BCFs to increase the challenges for bearing fault detection. The second case study analyses vibration signals from the CWRU dataset, especially those with combined outer race and inner race faults [37]. The third case uses the run-to-failure data from the IMS to assess the capability of the novel hybrid method in detecting early-stage naturally occurring faults in bearings. The final example uses signals from an accessory gearbox of a Safran jet engine to evaluate the effectiveness of the novel hybrid method in detecting bearing faults in a real application. This case is used in a Safran challenge, and the results from this study are compared with the best-published results [38]. The test scenarios of each dataset are summarised in Table 4.

### 4.1. Case Study 1: I2BS Sub-Scale Test Data

This case study uses bearing test data from an EU Clean Sky 2 Joint Undertaking under the European Union’s Horizon 2020 research and innovation programme. A sub-scale test rig was used to test smart bearings with multiple sensors under simulated aero-engine REB operations to develop intelligence in bearing health monitoring. The smart bearings are equipped with two piezoelectric acceleration sensors for vibration measurement, as well as a range of sensors for shaft and cage speeds, temperature, and strain on the outer race. The bearings for the sub-scale testing were three-point split inner race ball bearings. Key bearing and seeded fault dimensions as well as load and speed conditions are summarised in Table 5. Bearings with and without seeded faults on the outer race, inner race, and balls were tested under three different load and three different speed conditions. Each test was run for about 6 h. A shaker was used to add spectrum noise (up to 4 kHz) and specific frequencies, e.g., the BCFs with their harmonics to simulate aero-engine working conditions and challenge the detection strategy, i.e., identify what is a defect and what is noise. The vibration signals were sampled at 100 kHz frequency for 1 s duration at 30 s intervals throughout each test. 

**Figure 4:**
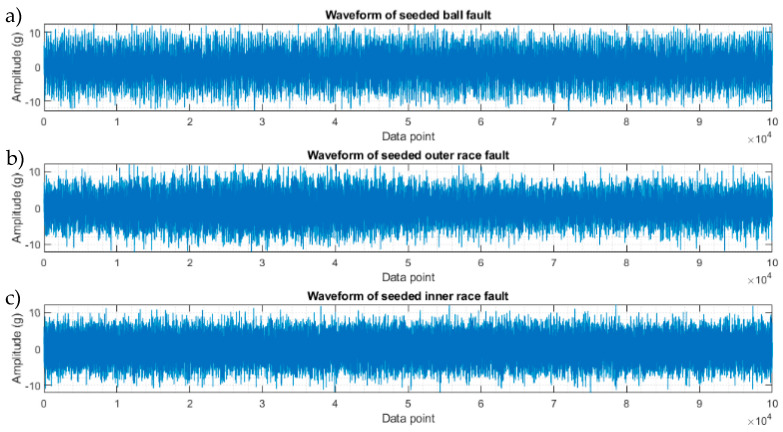
Waveforms of 1 s vibration data from three randomly selected I2BS sub-scale tests (**a**) with a ball defect of 0.4 mm indent at the shaft speed of 5000 rpm and axial load of 9 kN, (**b**) with an outer race defect 0.4 mm indent at the shaft speed of 5000, and (**c**) with an inner race defect 0.1 mm indent at the shaft speed of 5000.

**Figure 5:**
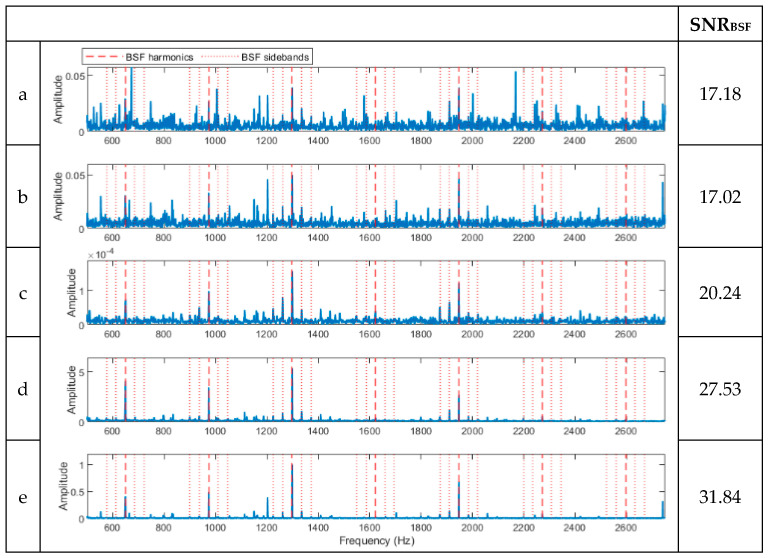
Spectra of the signal in Figure 4a processed using the five methods. (**a**) A spectrum of the full-band envelope. (**b**) A spectrum of the high-pass-filtered envelope. (**c**) A spectrum of the basic CPW envelope. (**d**) A spectrum of the ICPW envelope. (**e**) A spectrum of the novel hybrid method.

**Figure 6:**
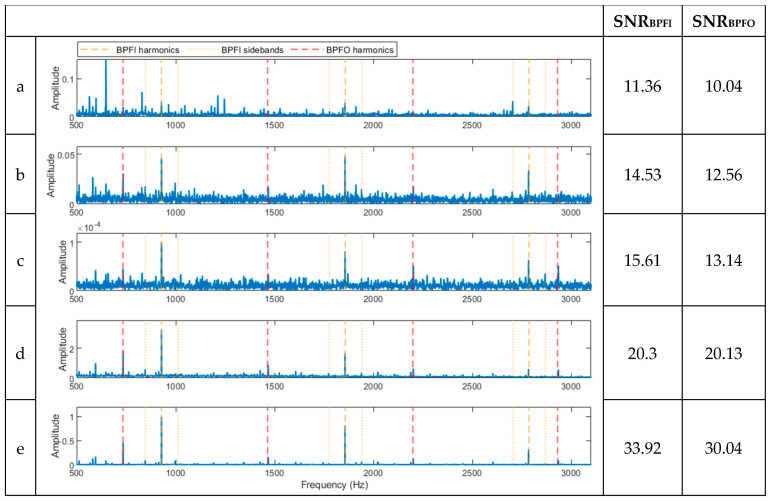
Spectra of the signal in Figure 4b processed using the five methods. (**a**) A spectrum of the full-band envelope. (**b**) A spectrum of the high-pass-filtered envelope. (**c**) A spectrum of the basic CPW envelope. (**d**) A spectrum of the ICPW envelope. (**e**) A spectrum of the novel hybrid method.

**Figure 7:**
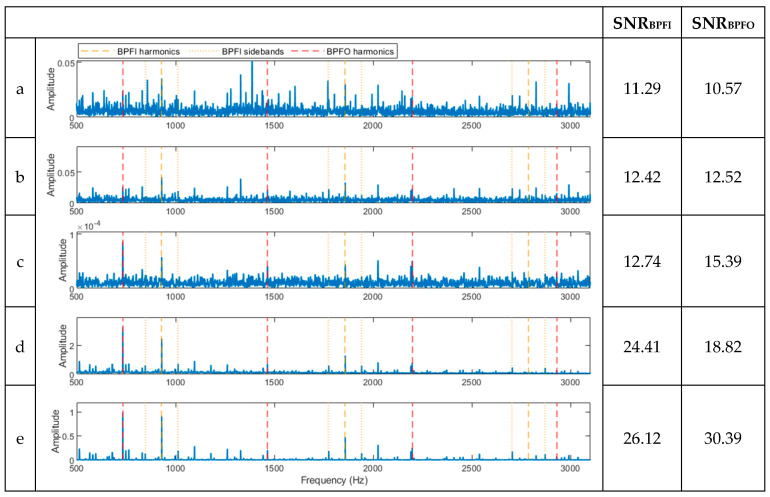
Spectra of the signal in Figure 4c processed using the five methods. (**a**) A spectrum of the full-band envelope. (**b**) A spectrum of the high-pass filtered envelope. (**c**) A spectrum of the basic CPW envelope. (**d**) A spectrum of the ICPW envelope. (**e**) A spectrum of the novel hybrid method.

Figure 4 shows three vibration signals selected at random from the I2BS tests, which were processed following the same procedure described in Section 3 for simulated signals, and the results are shown in Figure 5, Figure 6 and Figure 7. The noise level is considerably higher than that in the verification signals due to the high-speed conditions and the added noise during these tests. For the first signal (signal a) with a 0.4 mm ball defect, the full-band envelope and high-pass filtering methods, Figure 5a,b, are able to identify the BSFs but with very low SNRs. The basic CPW and ICPW detect the BSFs and some of the sidebands, Figure 5c,d; particularly, the ICPW with the higher SNR value is shown to be able to suppress the noise significantly (Figure 5d). This is due to the mathematical concept of the two methods, where CPW can remove periodic impulses. The spectrum obtained using the novel hybrid method, shown in Figure 5e, shows better detection, as well as higher SNR, of the BSFs and their sidebands than the other four methods.

The results for signal b, with a 0.4 mm outer race defect (Figure 4b), and signal c, with a 0.1 mm defect on the inner race (Figure 4c) are presented in Figure 6 and Figure 7, respectively, following the same analysis procedure. Again, the novel hybrid method demonstrated a better detection of the BCFs (with a higher SNR). It is also interesting to see that both BPFO and BPFI were detected with the method under each case due to the real and shaker noises. As the shaker applies impulses with a constant force, the BPFI harmonics in Figure 6e are not accompanied by sidebands. The results from signal c (Figure 7e) show that the novel hybrid method can successfully detect a very small inner race defect (BPFI and up to its third harmonics with sidebands), as well as the BPFO from the shaker. 

### 4.2. Case Study 2: CWRU Data

The CWRU vibration data have been widely used for bearing vibration signal processing method development in many studies. Bearing vibration signals were collected from a test rig that includes an electrical motor, a torque meter, and a dynamometer [37]. The test bearings were 6205–2RS JEM deep groove ball bearings. Single-point defects were seeded on the bearing outer race or the inner race using electro-discharge machining. The relevant dimensions of the bearing and the operating conditions are shown in Table 6.

**Figure 8:**
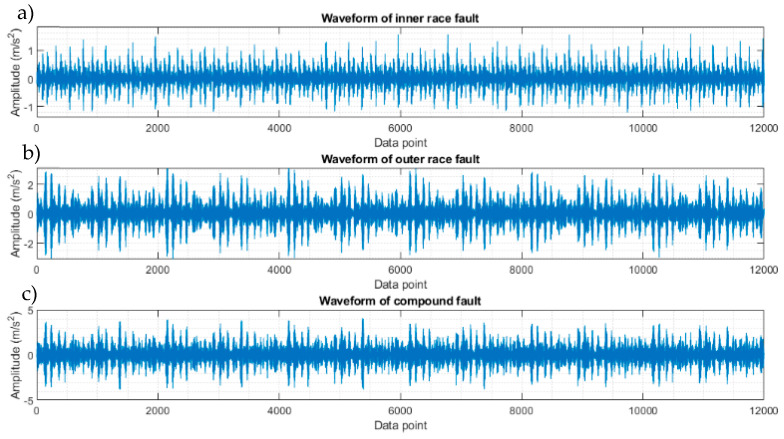
Initial and synthesised compound signals from CWRU data. (**a**) Waveform of a signal with an inner race fault, (**b**) waveform of a signal with an outer race fault, and (**c**) waveform of the compound signal.

**Figure 9:**
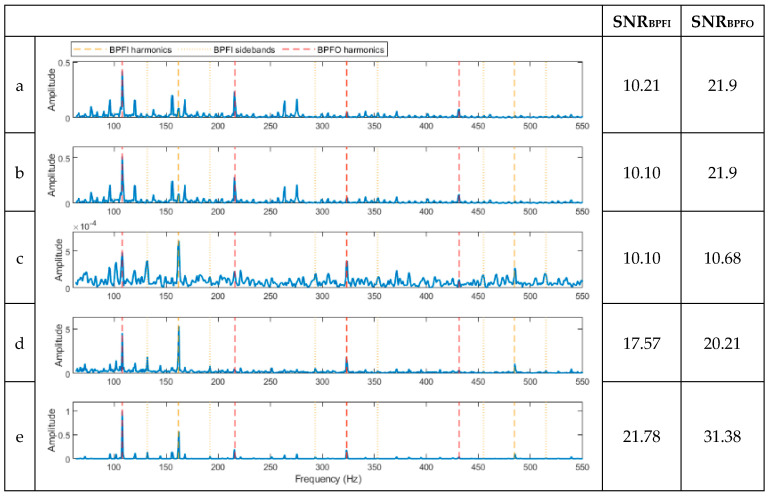
Results of the signal in Figure 8c after being processed using the various methods. (**a**) A spectrum of the full-band envelope. (**b**) A spectrum of the high-pass-filtered envelope. (**c**) A spectrum of the basic CPW envelope. (**d**) A spectrum of the ICPW envelope. (**e**) A spectrum of the novel hybrid method.

To evaluate the novel hybrid method, a signal with multiple faults is constructed by superimposing two separate signals of CWRU data, one from a bearing with an outer ring defect and the other with an inner ring defect at the smallest size in CWRU experimental data [25]. The waveforms of the signals with an individual fault and the synthesised signal are shown in Figure 8. The resulting spectra of the compound signal after being processed using the five techniques are shown in Figure 9. The full-band spectrum and high-pass filtered spectrum do not detect the inner ring damage (Figure 9a,b), while the basic CPW, ICPW, and the novel hybrid method can all detect both inner and outer ring faults, with the latter showing the highest SNR. (Figure 9c–e).

### 4.3. Case Study 3: IMS Run-to-Failure Test Data

**Figure 10:**
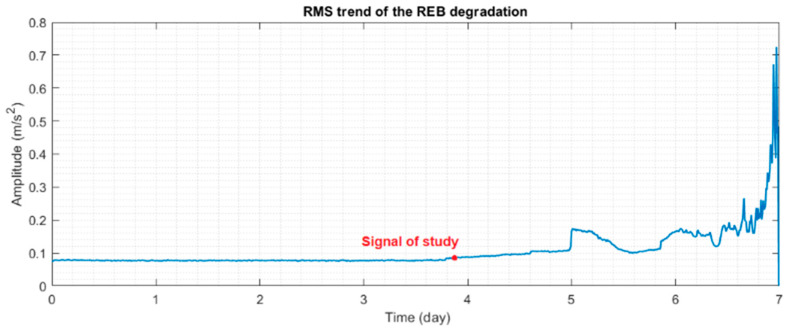
The root mean square (RMS) trend in the vibration signals acquired from the sensor located on the faulty bearing in the second test of the IMS.

This case aims to further evaluate the effectiveness of the novel hybrid method in detecting incipient bearing faults with the analysis of a run-to-failure bearing vibration signal from the IMS database at the University of Cincinnati [34]. The experimental test rig includes four double-row bearings from Rexnord ZA-2115 mounted on one shaft. Figure 10 shows the RMS vibration signal during the run-to-failure test, showing a point of interest where the raw vibration signal was analysed in this case study.

**Figure 11:**
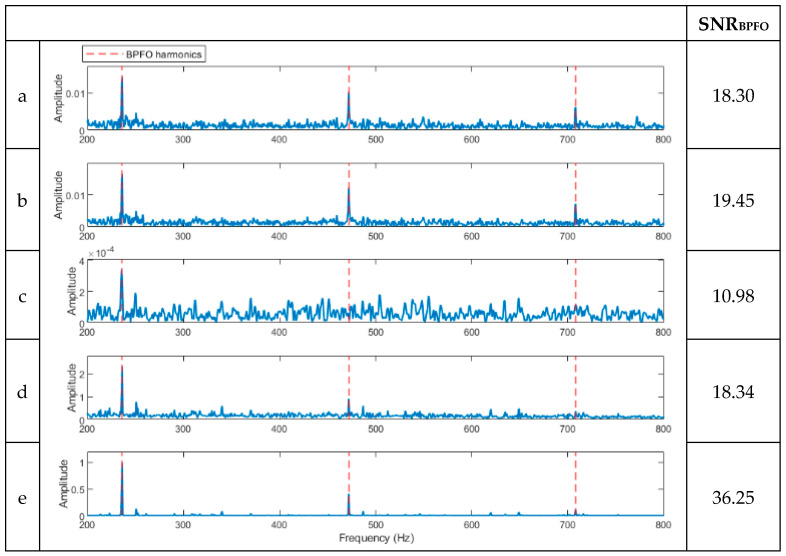
Diagnosis results of the signal of study in the early-stage bearing fault shown in Figure 10. (**a**) A spectrum of the full-band envelope. (**b**) A spectrum of the high-pass filtered envelope. (**c**) A spectrum of the basic CPW envelope. (**d**) A spectrum of the ICPW envelope. (**e**) A spectrum of the novel hybrid method.

As can be seen in Figure 11, the novel hybrid method can clearly detect the BPFO fault in the bearing at an early stage with a higher SNR than the full-band envelope and high-pass filter methods. Also, the CPW method only identifies the first harmonic, which lacks the necessary information for diagnostics. In contrast, the ICPW method detects two harmonics and therefore yields better results.

### 4.4. Case Study 4: Safran Jet Engine Test Data

**Figure 12:**
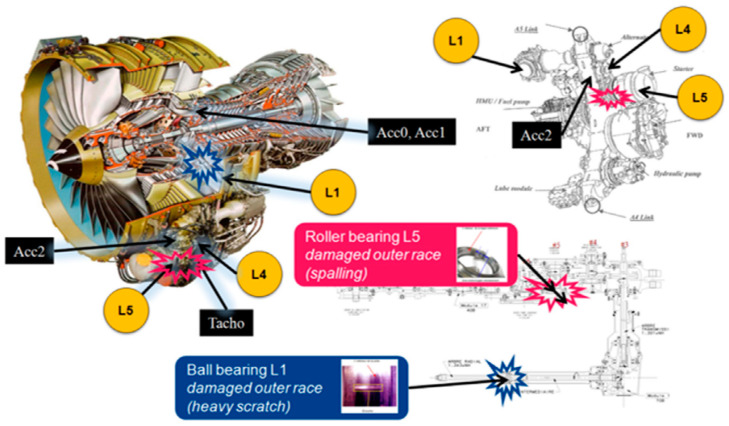
An overview of the jet engine and the accessory gearbox. Amber-coloured L labels identify shafts [38].

This evaluation is based on the dataset used for a Safran contest that took place during the Conference Surveillance 8, 20–21 October 2015, at the Roanne Institute of Technology, France. Vibration and tachometer signals were collected during a ground test campaign on a civil aircraft engine with two damaged bearings. This study focuses on the second exercise of the contest regarding the diagnosis of a faulty bearing. Signals from two accelerometers and one tachometer, named “Acc1”, “Acc2”, and “Tacho”, were recorded during a slow acceleration, from idle to full power [38]. Two faults were identified in the contest, including an outer ring fault and a cage fault in the REB on shaft L5 in the accessory gearbox (Figure 12). 

**Figure 13:**
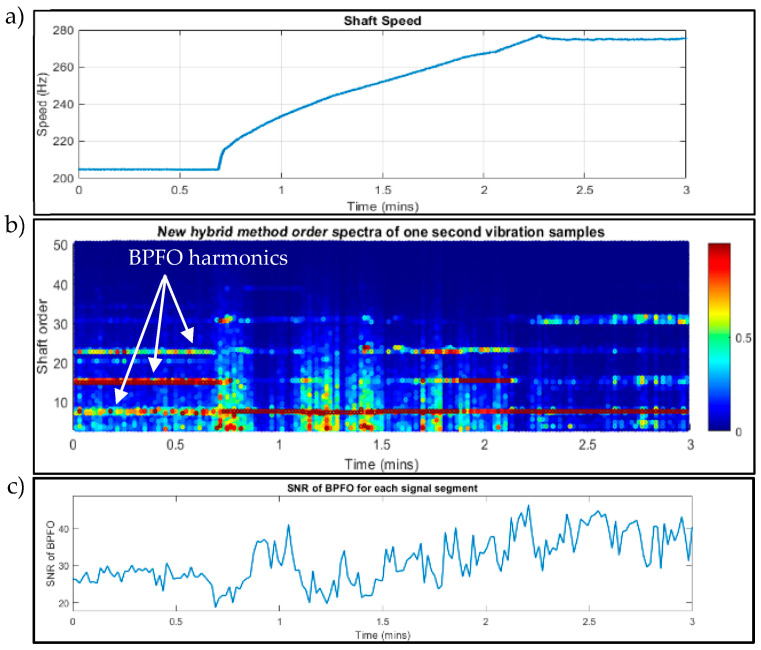
Data analysis of Acc2 and Tacho sensing systems. (**a**) The instantaneous angular speed of the shaft by converting the Tacho signal to speed. (**b**) Colourmap (with a window length of 1 s or 50,000 data points) of order spectra of Acc2 vibration samples using the novel hybrid method. (**c**) SNR values of the BPFO in each signal segment.

Detailed information regarding sensors, bearing type, and engine test conditions can be found in [38]. By analysing the signals from Acc2 (note: the analysis of signals from Acc1 does not show any bearing faults, which is in line with other research methods [38]) and converting the Tacho sensor impulses to speed, Figure 13a shows the speed graph during the test.

Instead of computing order tracking (COT), in this study, the 3 min vibration signal is divided into 180 segments to reduce the effect of speed changing and enhance bearing fault diagnosis. The novel hybrid method is used on the 180 segments with a MATLAB program, and a colour map of the spectra is shown in Figure 13b. An outer ring fault is clearly evidenced by the persistent BPFO and its harmonics throughout the whole duration (BPFO first harmonic order = 7.75) during both the steady state and run-up operating conditions. Moreover, the SNR values of the BPFO in Figure 13c indicate the effectiveness of the proposed method, which has an average SNR_BPFO_ of 35.36 (minimum of 19.99 and a maximum of 47.04).

**Figure 14:**
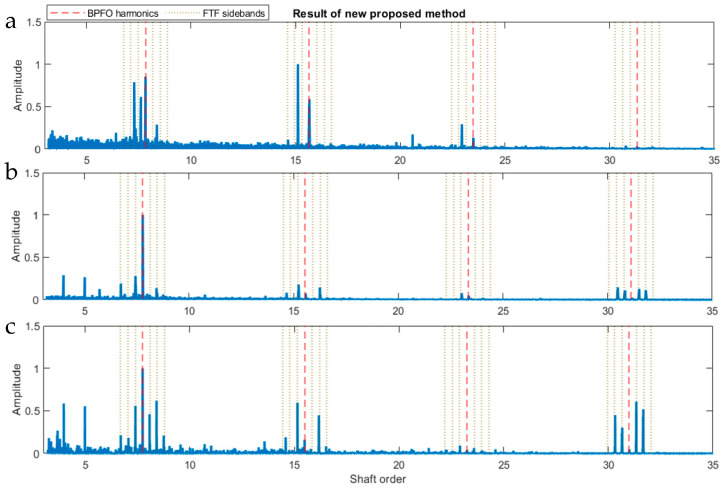
Diagnosis results using the novel hybrid method. (**a**) First 40 s. (**b**) Last 30 s. (**c**) One-second segment at 2.8 min.

However, the cage fault is not visible in any segment or at any time in Figure 13b. For further analysis, several segments with different durations were analysed separately. Among these segments, two long segments, one from the first 40 s and the other from the last 30 s, and one short segment of 1 s at 2.8 min were sampled from the original signal. The results of the analysis of these three signals are shown in Figure 14. All three spectra show one or more BPFO harmonics, and also in Figure 14b,c, a number of FTF sidebands exist around them. In some cases, sidebands are visible without BPFO harmonics (Figure 14b,c, fourth harmonics). Comparing the three spectra, it appears that the 2.8 min sample contains the strongest FTF sidebands, indicating the cage fault may not exist at the beginning but only developed at a later stage of the test. This demonstrates that the novel hybrid method can detect both faults in a noisy engine environment under transient conditions without the requirement of additional pre-processing such as COT that many other techniques need.

## 5. Comparison of the Novel Hybrid Method with Fast Kurtogram

Fast Kurtogram (FK) is a widely accepted method for bearing fault diagnosis [10]. Thus, a comparison between the novel hybrid method and FK is conducted here to demonstrate the effectiveness of the novel hybrid method. In order to ensure a fair comparison, when conducting the Fast Kurtogram (FK) analysis for the selection of an appropriate filtering band during the fault diagnosis process, up to 10 levels are systematically explored. The choice of filtering band in FK analysis is contingent on its suitability for the given application, and this suitability is often reflected in the level number chosen for fault diagnosis.

The results from the FK analysis of the experimental data from three sources, including I2BS, CWRU, and IMS, are summarised in Table 7, illustrating the SNR values of the known and detected BCFs with the novel hybrid method (third column) and those detected with FK at levels 1 to 5. The FK results above level 5 are not shown as no BCFs are detected from levels 6 to 10. The spectral results from this analysis can be found in the Appendix A and Appendix B, providing details of the BCF detection.

As can be seen in Table 7, at different levels, FK detected different BCFs even for the same signal except for the first signal from I2BS, i.e., the ball fault. For the second signal, where the novel hybrid method detected both the BPFO (due to a seeded fault) and the BPFI (from the shaker), only FK level 2 detected both, while the other four levels either detected nothing (levels 1, 4, and 5) or only BPFO (level 3), which demonstrate the challenges FK (and other similar techniques) face in selecting the most suitable filtering band for bearing fault detection and diagnosis.

**Figure A5:**
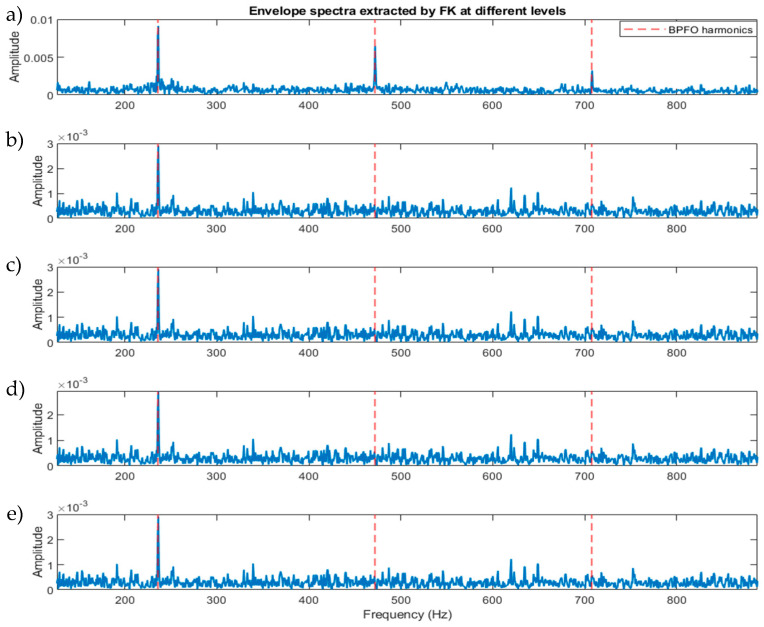
FK envelope spectra of the IMS data up to different levels. (**a**) First level, (**b**) second level, (**c**) third level, (**d**) fourth level, and (**e**) fifth level.

**Figure A6:**
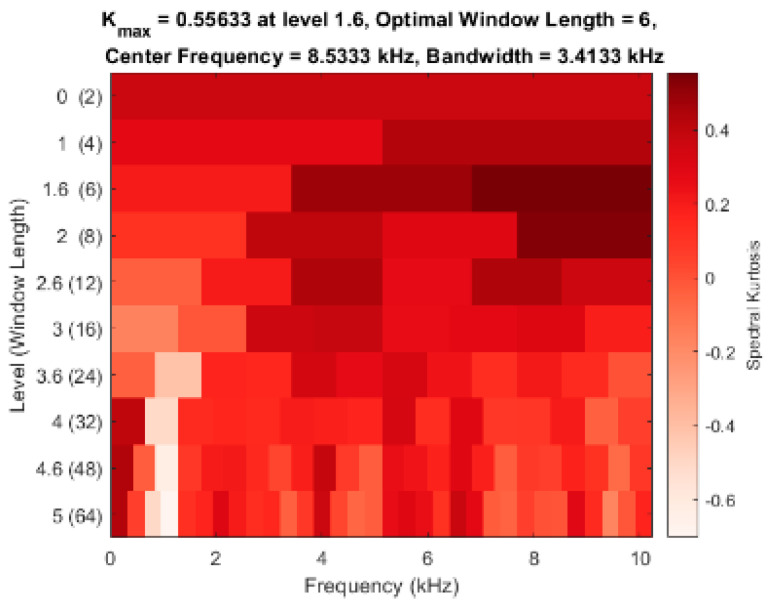
FK frequency map results of IMS data up to level 5.

To give an example of the limitation of the FK method, the envelope spectra of FK analysis on IMS data up to level 5 is shown in Figure A6. As can be seen at up to level 1, the highest kurtosis index lies in the band of 5.1–10.02 kHz, which is used for the envelope analysis in Figure A5a. This is shown to detect the expected OR fault. Looking at the map in Figure A6, at up to level 5, the highest kurtosis index changes to the band of 6.8–10.02 kHz. Using this filter band at levels 3–5 produced the results shown in Figure A5b–e, which have limited diagnostic. Hence, the kurtosis index-based filter band selection is not reliable in these cases.

**Figure A7:**
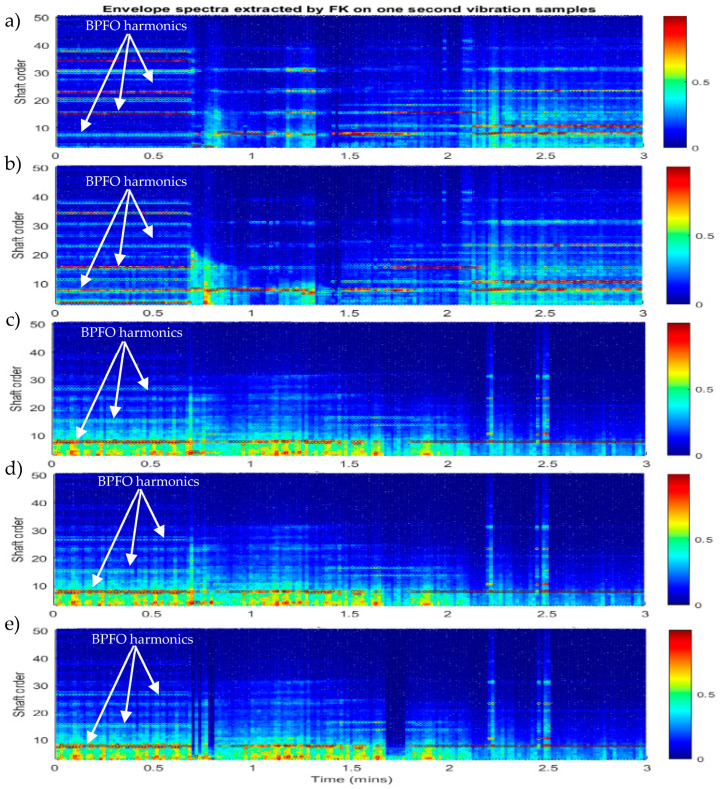
Colourmap (with a window length of 1 s or 50,000 data points) of order spectra of Acc2 vibration samples using FK method up to different levels. (**a**) First level, (**b**) second level (**c**) third level, (**d**) fourth level, and (**e**) fifth level.

The FK analysis was also applied to the Safran jet engine data, and the results are presented in Figure A7, where up to level 5 of FK are explored. Also included are the SNR results presented in Table 8. Since the Safran jet engine data were analysed based on signal partitioning, the SNR results are shown based on the minimum, average, and maximum values of the 180 signal segments at each FK level. While similar results are observed at all levels, it should be noted that no level consistently highlights the BPFO harmonics in all segments. As indicated in Table 8, within each level, some segments exhibit relatively low SNR values, signifying that BPFO harmonics could not be effectively emphasised using the FK method in these particular segments. Also, unrelated harmonics are seen, especially at the first and second levels, as shown in Figure A7. 

In summary, compared with the novel hybrid method, the FK method, although it can detect BCFs correctly under most circumstances, requires an optimisation of the level selection to ensure the most suitable filter band is used to avoid the misidentification of BCFs. Furthermore, the use of the kurtosis index as the fault indicator may provide unreliable outcomes at times. 

## 6. Conclusions

This paper presented a novel hybrid method for bearing fault diagnosis based on ICPW and high-pass-filtering methods. The novel hybrid method is built on two approaches that are commonly used for narrowband and wideband frequency analysis. It demonstrated improved bearing fault detection and diagnosis, especially when compared with existing methods such as HFRT and FK. By eliminating the requirement for the manual selection of filtering bands, accurate and automated bearing fault diagnosis is feasible. The novel hybrid method was verified with the analysis of numerically simulated signals containing known BCFs, gear mesh frequencies, shaft harmonics, and white noise and validated using experimental data from three different test sources and data from a real jet engine, encompassing a wide range of bearing and test operating conditions. Furthermore, a Signal-to-Noise Ratio (SNR) metric was defined to quantify the BCF detection capability of the various methods, including the full-band envelope, high-pass filtered envelope, CPW envelope, ICPW envelope, FK method, and novel hybrid method. The SNR metrics, alongside the results of computing time analysis presented in Table A1 of the Appendix B, collectively demonstrate the effectiveness of the novel hybrid methodology. 

In summary, the main outcomes of this work are:

- A simulated signal was used to replicate bearing OR and IR faults, while also accounting for shaft and gear mesh harmonics. The novel hybrid method detected both bearing faults and GMFs, providing enhanced clarity in fault detection. None of the other analysis methods investigated were able to detect the bearing faults and gear mesh frequencies.
- The effectiveness of the novel hybrid method was demonstrated with an analysis of two separate experimental seeded fault tests. These studies, namely, I2BS (with fault sizes of 0.1 mm and 0.4 mm, along with a spectrum noise) and CWRU (featuring the smallest fault size of 0.177 mm), highlighted the robustness of the developed method. The method successfully detects small defect sizes and identifies concurrent multiple faults by highlighting their corresponding bearing BCFs.
- The run-to-failure bearing data obtained from the IMS test was analysed and showed the effectiveness of the developed method in detecting faults at an early stage. By monitoring the RMS trend of the data, a vibration signal was selected at the beginning of a small increase in the RMS value. The novel hybrid method subsequently identified the BCFs, which is evidence of its usefulness for early-stage fault diagnosis.
- In the final case study, the method was evaluated with its application to real-world Safran jet engine data obtained during the ground testing of a civil jet engine under transient run-up speed conditions. The data were collected in a noisy operating environment: a difficulty highlighted in similar research endeavours. The developed method was able to find bearing faults in all data segments without requiring COT. This success highlights the novel hybrid method’s performance under challenging operating conditions.

This study also shows the limitation inherent in the FK method pertaining to filter band selection using kurtosis value analysis. In contrast, the new method obviates the FK need for band level selection, parameter adjustment, or optimization and successfully highlights bearing faults within a clear and uncluttered spectrum with a minimal impact of background noise. This was demonstrated with the quantifiable SNR analysis results that the choice of the level number in FK can significantly impact SNR values (e.g., in Table 7, the SNR values of 8.36 and 18.06 are observed for level one and level two, respectively, for the OR characteristic frequencies in I2BS_OR_). Also, when an optimal level number is predetermined for the FK method, the novel hybrid method outperforms the FK method (e.g., the SNR of 36.25 and 17.72 achieved with the novel hybrid and the first level of the FK methods for IMS data, shown in Table 7). Due to the normalisation process in the hybrid method conducted at several stages, as shown in Figure 1, the severity of detected bearing faults may not be truly reflected. Therefore, further investigation is being conducted for fault severity assessment.

Future work will focus on implementing this method as a tool for generalised fault detection for REBs of different configurations and under a wide range of operating conditions. The method will also be further developed using machine learning techniques. The potential of this approach is to overcome the need for large amounts of training data when machine learning models are transferred from one application to another.

## Figures and Tables

**Table 1:** Parameters used in the simulated vibration.

α	$f_{n},kHz$	$A_{b}$	$A_{s}$	$A_{g}$	$N_{g}$	$\varphi_{s}$	$\varphi_{g}$
0.05	2, 4, 6, 8	0.25, 0.5, 0.75, 1	3	2.5	6	0–2π	0–2π

**Table 2:** Operating condition, sampling rate, and fault frequencies in IMS experiments.

Radial Load, kN	$f_{shaft},\;Hz$	$f_{sampling\_rate},\;Hz$	$f_{BPFI},$ Hz	$f_{BPFO},$ Hz
26.7	33.34	20,480	296.9	236.4

**Table 3:** REB model and dimensions used in IMS experiments.

REB Model	$D_{pitch}\;(mm)$	$D_{ball}\;(mm)$	Z	∅ (deg)
Rexnord ZA-2115	71.5	8.4	16	15.71

**Table 4:** Test scenarios of the investigated case studies.

Case Studies	Case Scenarios
I2BS	Seeded faults were created on the bearing components, and a shaker was used to introduce spectrum noise and bearing BCFs to simulate aero-engine operating conditions. The data were collected under three steady-state operating conditions.
CWRU	Seeded faults were created on the bearing components. The data were collected under one steady-state operating condition.
IMS	Run-to-failure bearing degradation test at one steady-state operating condition.
Safran jet engine	Jet engine ground test with real faults run at steady-state and transient (run-up speed) operating conditions.

**Table 5:** I2BS bearing dimensions and test conditions.

$D_{pitch}\;(mm)$	$D_{ball}\;(mm)$	Z	∅ (deg)	$f_{shaft}\;(rpm)$	Axial Load (kN)	Seeded Defect Diameter (mm)
75	9.525	20	15	5000, 10,000, 14,000	1, 2.5, 9	0.1, 0.2, 0.4

**Table 6:** Bearing dimensions and test conditions of the CWRU signals selected for this study.

$D_{pitch}$ (mm)	$D_{ball}$ (mm)	Z	∅ (deg)	$f_{shaft}$ (rpm)	Load (kN)	$f_{sampling\;rate}$ (kHz)	Seeded Defect Diameter (mm)
44	8.2	9	0	1730	2.23	12	0.177

**Table 7:** A summary of the diagnosis results using the FK method.

Data Source	Available BCFs	SNR of the Available BCFs Detected with the Novel Hybrid Method	SNR of the Available BCFs Detected with FK				
			Level 1	Level 2	Level 3	Level 4	Level 5
**I2BS_Ball_**	**Ball**	31.84	20.25	17.86	19.46	19.46	19.46
**I2BS_OR_**	IR	33.92	10.70	13.12	7.30	8.62	8.62
	OR	30.04	8.36	18.06	13.75	8.02	8.06
**I2BS_IR_**	IR	26.12	11.14	15.91	15.91	9.15	8.43
	OR	30.39	11.19	17.34	17.34	23.07	19.21
**CWRU**	IR	21.78	7.38	13.60	9.06	9.06	9.06
	OR	31.38	20.49	17.34	16.44	16.44	16.44
**IMS**	OR	36.25	17.72	14.58	14.58	14.58	14.58

**Table 8:** SNR_BPFO_ values of FK results at up to level 5 in Safran jet engine bearing data. The graph on the left shows the SNR_BPFO_ variations within 3 min for the five levels. A summary of the SNR_BPFO_ is presented in the table on the right.

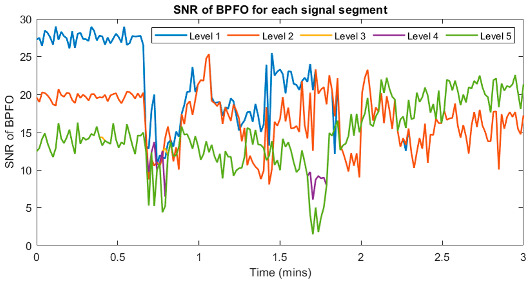	**Level Number**	**SNR_BPFO_**		
		**Minimum**	**Average**	**Maximum**
	1	10.98	22.03	29.41
	2	8.16	18.47	25.73
	3	8.06	16.17	22.72
	4	6.60	16.15	22.72
	5	1.79	16.02	22.72

**Figure A1:**
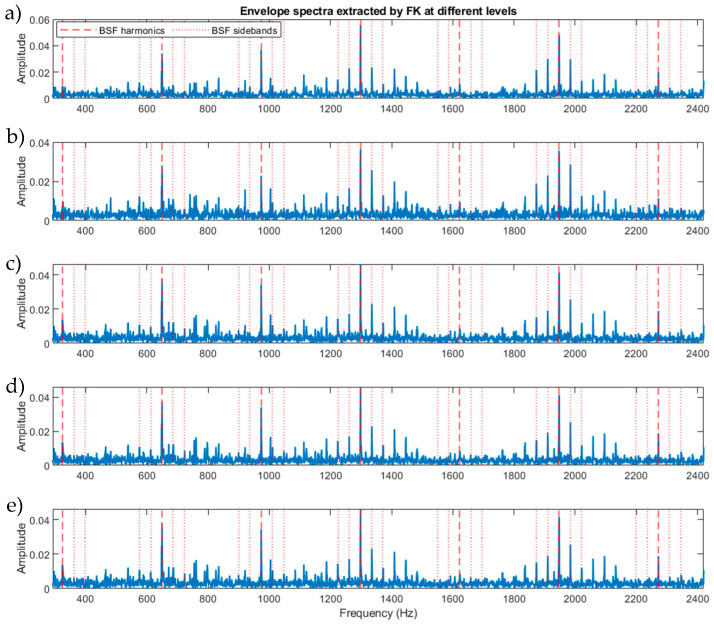
FK envelope spectra of the I2BS data with a ball fault up to different levels. (**a**) First level, (**b**) second level, (**c**) third level, (**d**) fourth level, and (**e**) fifth level.

**Figure A2:**
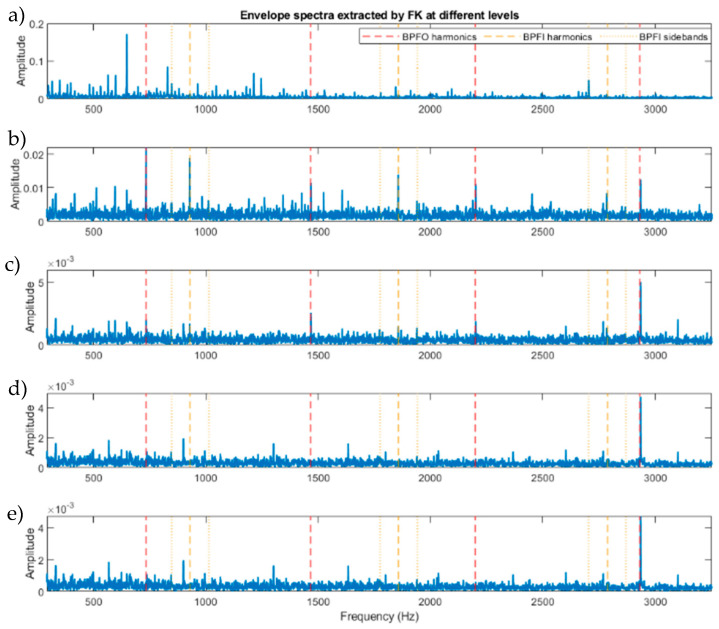
FK envelope spectra of the I2BS data with an OR fault up to different levels. (**a**) First level, (**b**) second level, (**c**) third level, (**d**) fourth level, and (**e**) fifth level.

**Figure A3:**
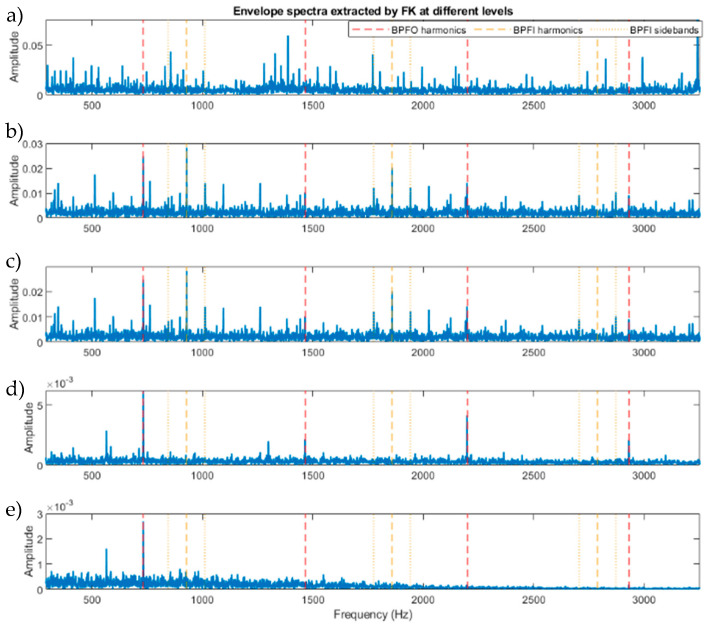
FK envelope spectra of the I2BS data with an IR fault up to different levels. (**a**) First level, (**b**) second level, (**c**) third level, (**d**) fourth level, and (**e**) fifth level.

**Figure A4:**
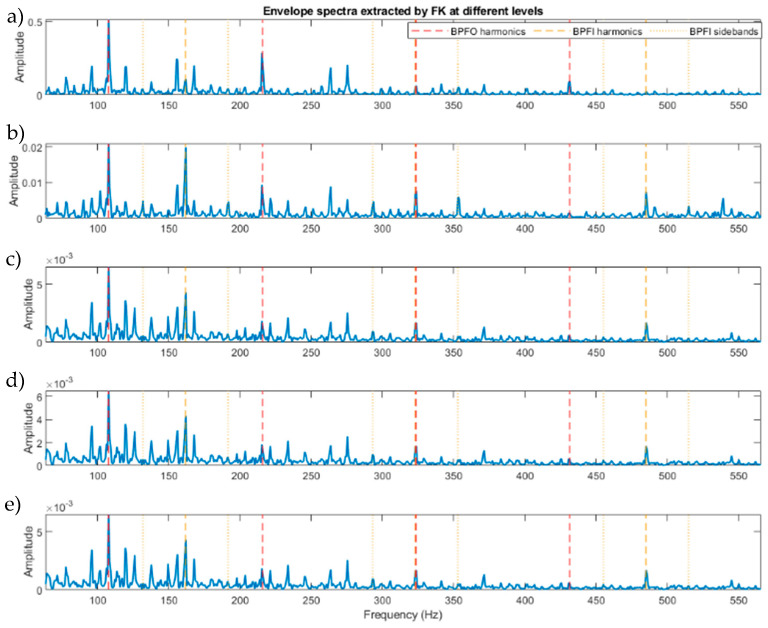
FK envelope spectra of the CWRU data up to different levels. (**a**) First level, (**b**) second level, (**c**) third level, (**d**) fourth level, and (**e**) fifth level.

## Data Availability

I2BS data has been collected in our project and study. Other experimental data used in this study, are available in public domain of the literature.

## Appendix B

It is important to confirm that the computing time of the novel hybrid method is comparable to or faster than commonly used methods (see data shown in Table 7). The computing time for processing one sample of the 1 second data from simulated signals and experimental data in MATLAB R2022a software on a laptop with an Intel (R) Core (TM) i7-10850H CPU and 16 GB RAM is less than 3 s, while for the 180 s engine data, it increases to 25.6 s. As it was explained in Section 2.2, the novel hybrid method performs bandpass filtering with the bandwidth of the 5 × BPFI of the target bearing. As the duration of the filtering process depends on how many filters are applied, the computing time will change if these parameters are altered.

**Table A1:** Computing time duration for the data used in this study.

Data Name	Signal Duration Time (s)	Processing Time (s)
Sub-scale I2BS	1	2.67
Safran _colourmap_	180	25.6
Safran _1 second segment_	1	0.31
IMS	1	0.76
CWRU	1	0.25
